# Supplementary material for: Unique Salt-Tolerance-Related QTLs, Evolved in Vigna riukiuensis (Na+ Includer) and V. nakashimae (Na+ Excluder), Shed Light on the Development of Super-Salt-Tolerant Azuki Bean (V. angularis) Cultivars
Source: Plants (Basel). 2023 Apr 17;12(8):1680. doi: 10.3390/plants12081680 (PMC10146836; doi:10.3390/plants12081680)
Supplement: Supplementary file 1 [file plants-12-01680-s001.zip › plants-2291851-supplementary.pdf]

Table S1. RAD markers from *V. nakashimae* 'Ukushima', *V. riukiuensis* 'Tojinbaka' and F_2 population.

	Number of reads	Mapping ratio to genome (%)	Number of RAD markers			Mean coverage depth[*2]		
			Total	Chr1-11	Scaffolds	Total	Chr1-11	Scaffolds
All samples	536,785,685		25,350[*3]					
V. nakashimae 'Ukushima'	13,052,980[*1]	74.1	15,438[*4]	14,856	582	36.8	36.1	54.2
V. riukiuensis 'Tojinbaka'	6,632,164	62.0	5,469[*5]	5,348	121	32.4	32.0	41.3
F_2 population (310 plants)	515,437,838							
Average per plant	1,662,703	55.4	2,485[*6]	2,485	19	17.4	17.4	15.3

[*1] The custom genome constructed using 'Ukushima' RAD-tag reads.
[*2] Mean read depth per SNP sites.
[*3] Total detected SNPs (filtered by depth and quality)
[*4] SNPs of homozygous A (same to reference) in 'Ukushima'.
[*5] SNPs of homozygous B in 'Tojinbaka'.
[*6] SNPs of homozygous A and B in 'Ukushima' and 'Tojinbaka', A or B or H in F_2 plants.

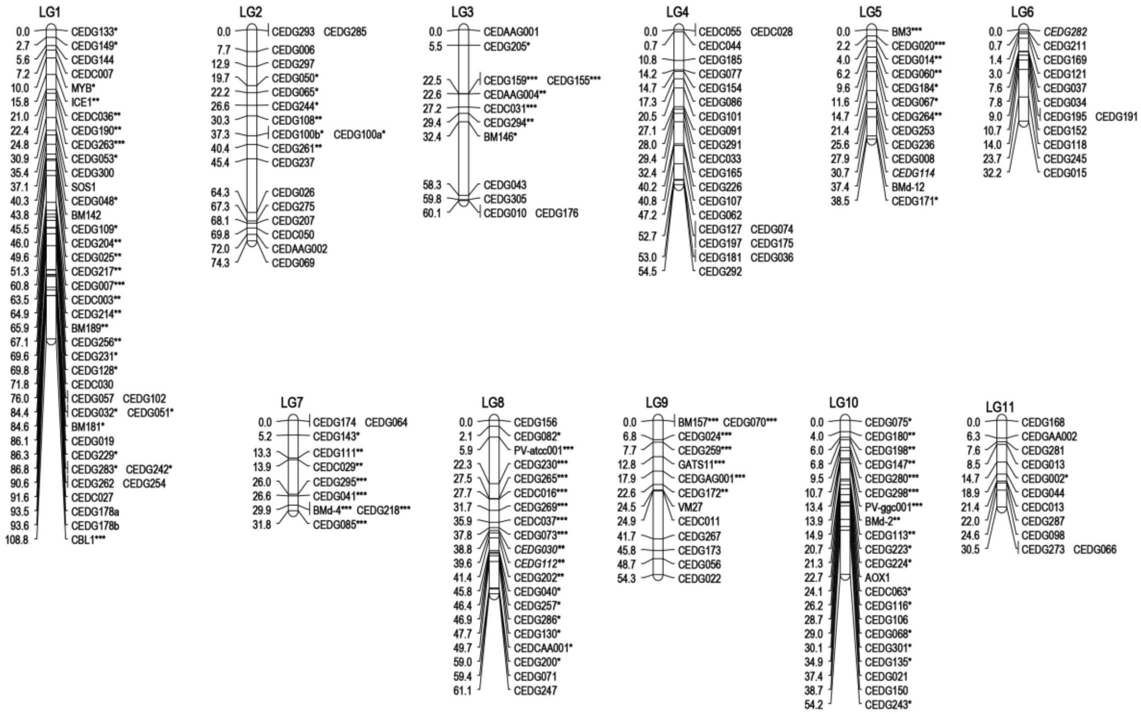

Figure S1. linkage map of population A

A genetic map of interspecific cross constructed from an F_2 population (*V. angularis* 'Kyoto Dainagon' x *V. riukiuensis* 'Tojinbaka'). Map distances and marker names are shown on the left and right side of the linkage groups, respectively. Marker names in italic indicate dominant loci. Markers showing significant deviation from the expected segregation ratio at the 0.05, 0.01 and 0.001 levels are indicated with *, ** and ***, respectively.

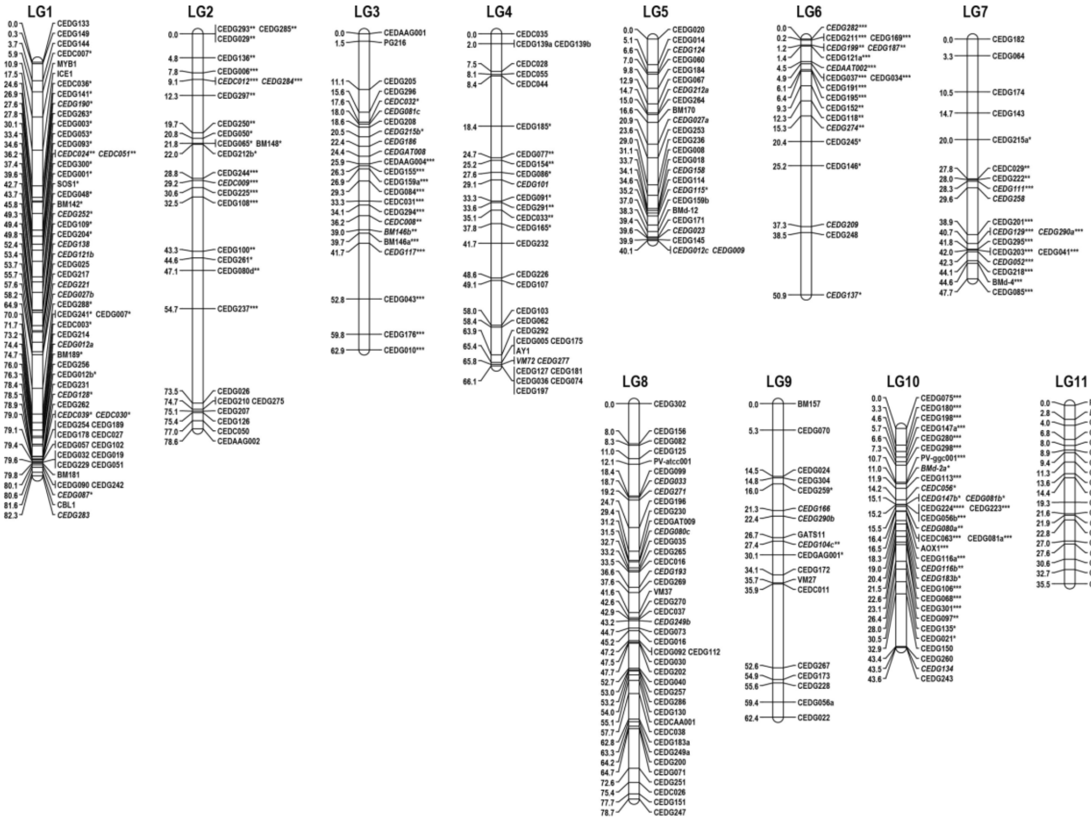

Figure S2. linkage map of population B

A genetic map of interspecific cross constructed from an F_2 population (*V. angularis* 'Kyoto Dainagon' x *V. nakashimae* 'Ukushima'). Map distances and marker names are shown on the left and right side of the linkage groups, respectively. Marker names in italic indicate dominant loci. Markers showing significant deviation from the expected segregation ratio at the 0.05, 0.01 and 0.001 levels are indicated with *, ** and ***, respectively.

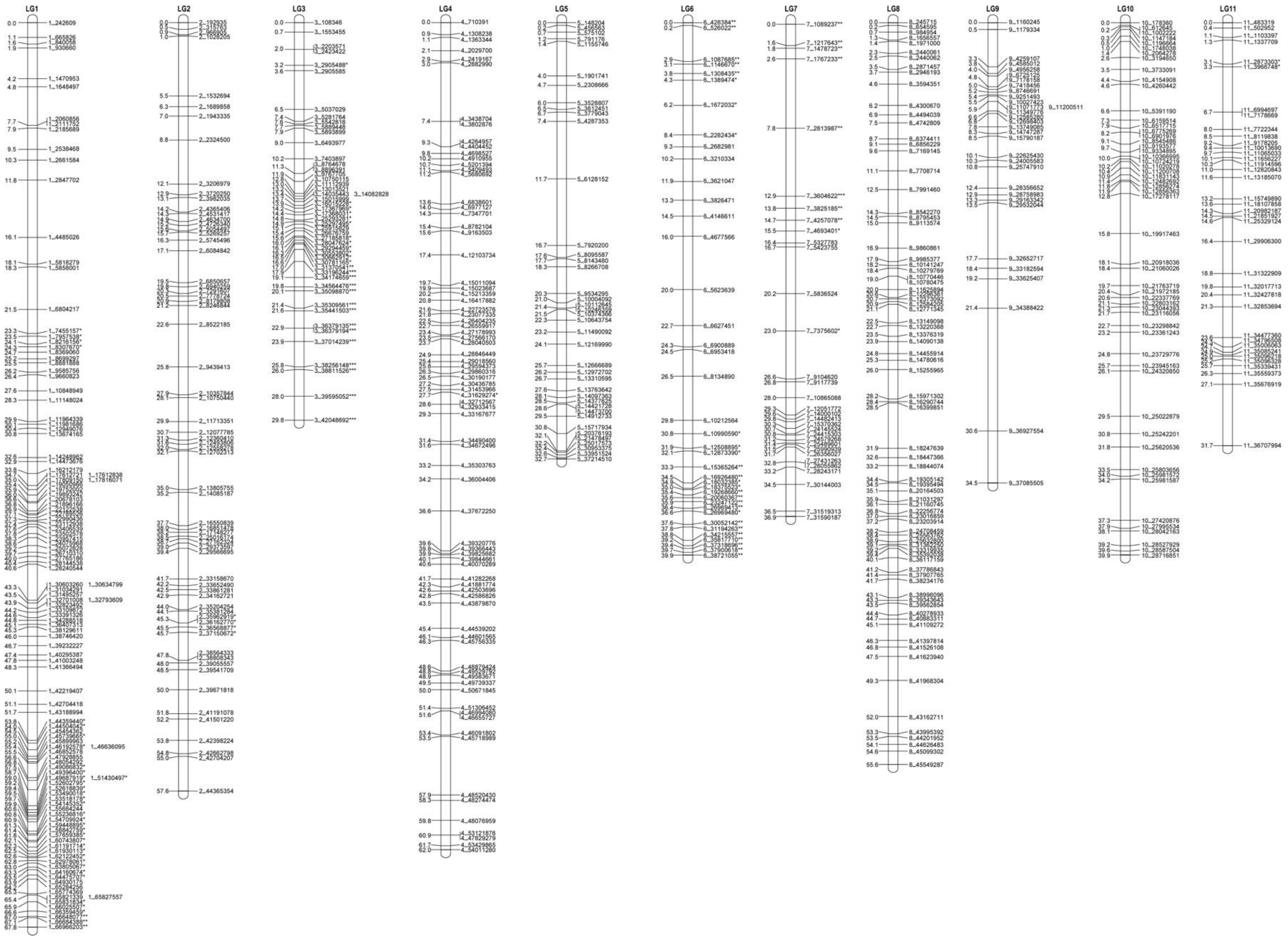

Figure S3. linkage map of population C

A genetic map of interspecific cross constructed from an F_2 population (*V. nakashimae* 'Ukushima' x *V. riukiuensis* 'Tojinbaka'). Map distances and marker names are shown on the left and right side of the linkage groups, respectively. Marker names in italic indicate dominant loci.

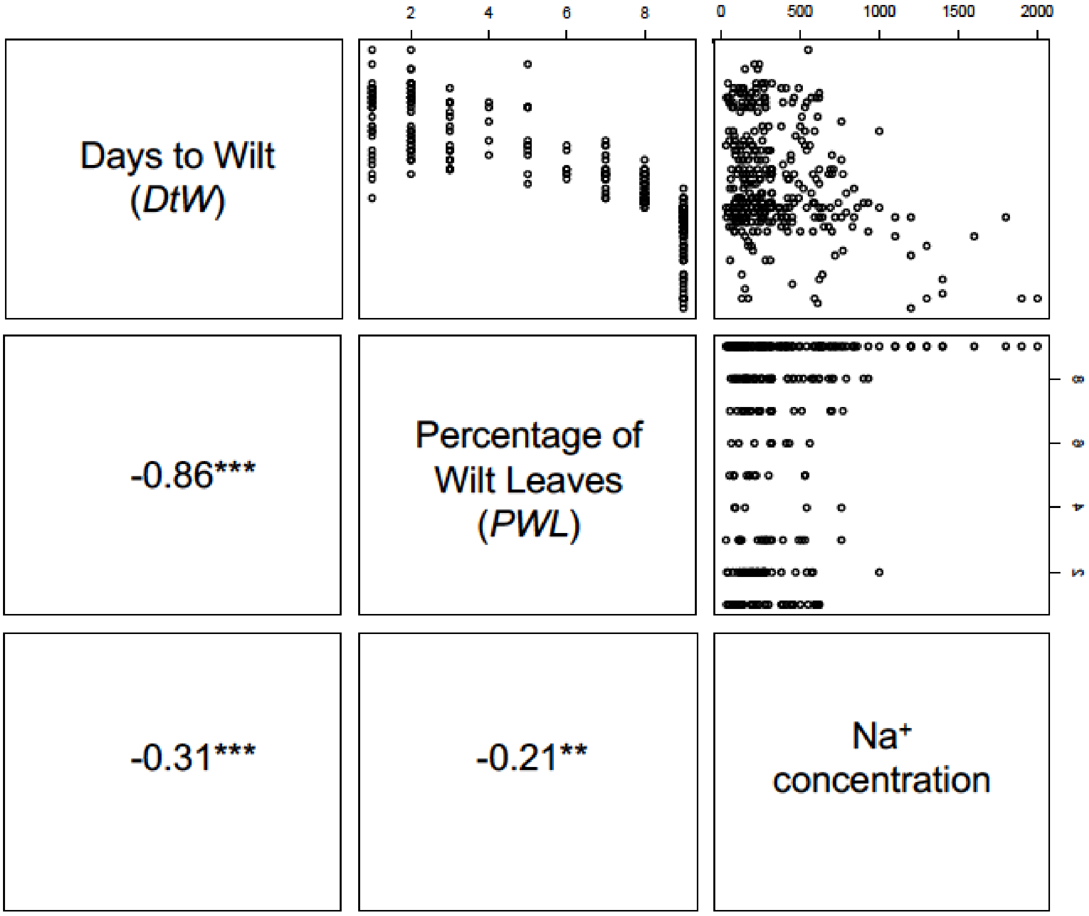

Figure S4. Correlations among traits in population C.

(Upper right) The correlation among Days to Wilt (*DtW*), Percentage of Wilt Leaves (*PWL*) and Na^+ concentration in primary leaf of F_2 population derived from a cross between *V. nakashimae* 'Ukushima' x *V. riukiuensis* 'Tojinbaka'. (Bottom left) Pearson's product-moment correlation coefficient. (*** P<0.0001, **P<0.001, *P<0.01)

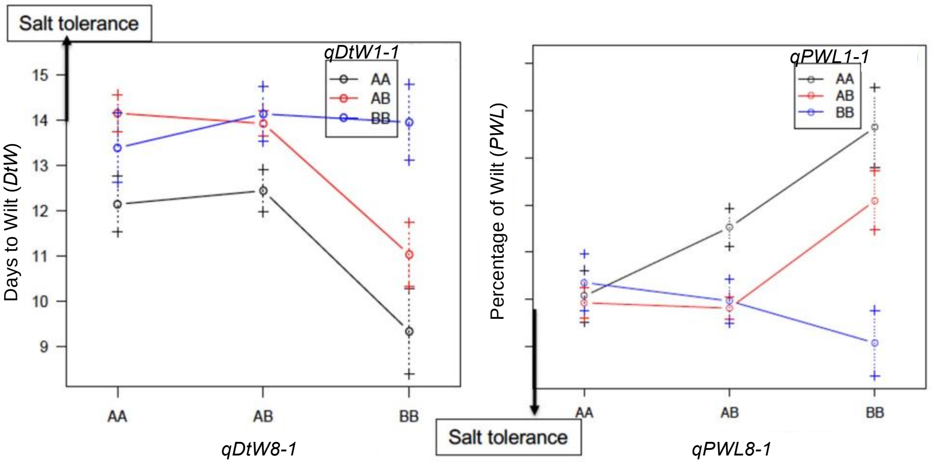

Figure S5. Genetic interaction

Genetic interaction was observed in (left)Day to Wilt (*DtW*) and (right)Percentage of Wilt Leaves (*PWL*) between *qDtW1-1/qPWL1-1* (CEDG242, BM181) and *qDtW8-1/qPWL8-1* (PV-atcc001) in population A (*V. angularis* 'Kyoto Dainagon' [genotype: A] x *V. riukiuensis* 'Tojinbaka' [genotype: B]).
